# Modulation of ipsilateral motor evoked potentials during bimanual coordination tasks

**DOI:** 10.3389/fnhum.2023.1219112

**Published:** 2023-09-06

**Authors:** Miriam Altermatt, Harry Jordan, Kelly Ho, Winston D. Byblow

**Affiliations:** ^1^Neural Control of Movement Lab, Department of Health Sciences and Technology, ETH Zürich, Zürich, Switzerland; ^2^Movement Neuroscience Laboratory, Department of Exercise Sciences, The University of Auckland, Auckland, New Zealand; ^3^Clinical Neuroscience Laboratory, Department of Medicine, The University of Auckland, Auckland, New Zealand; ^4^Centre for Brain Research, The University of Auckland, Auckland, New Zealand

**Keywords:** ipsilateral motor control, motor evoked potentials (MEPs), TMS, cooperative hand movements, reticulo-spinal control

## Abstract

**Introduction:**

Ipsilateral motor evoked potentials (iMEPs) are difficult to obtain in distal upper limb muscles of healthy participants but give a direct insight into the role of ipsilateral motor control.

**Methods:**

We tested a new high-intensity double pulse transcranial magnetic stimulation (TMS) protocol to elicit iMEPs in wrist extensor and flexor muscles during four different bimanual movements (cooperative—asymmetric, cooperative—symmetric, non-cooperative—asymmetric and non-cooperative—symmetric) in 16 participants.

**Results:**

Nine participants showed an iMEP in the wrist extensor in at least 20% of the trials in each of the conditions and were classified as iMEP^+^ participants. iMEP persistence was greater for cooperative (50.5 ± 28.8%) compared to non-cooperative (31.6 ± 22.1%) tasks but did not differ between asymmetric and symmetric tasks. Area and amplitude of iMEPs were also increased during cooperative (area = 5.41 ± 3.4 mV × ms; amplitude = 1.60 ± 1.09 mV) compared to non-cooperative (area = 3.89 ± 2.0 mV × ms; amplitude = 1.12 ± 0.56 mV) tasks and unaffected by task-symmetry.

**Discussion:**

The upregulation of iMEPs during common-goal cooperative tasks shows a functional relevance of ipsilateral motor control in bimanual movements. The paired-pulse TMS protocol is a reliable method to elicit iMEPs in healthy participants and can give new information about neural control of upper limb movements. With this work we contribute to the research field in two main aspects. First, we describe a reliable method to elicit ipsilateral motor evoked potentials in healthy participants which will be useful in further advancing research in the area of upper limb movements. Second, we add new insight into the motor control of bimanual movements. We were able to show an upregulation of bilateral control represented by increased ipsilateral motor evoked potentials in cooperative, object-oriented movements compared to separate bimanual tasks. This result might also have an impact on neurorehabilitation after stroke.

## Introduction

Most activities of daily living are performed bimanually (Bailey et al., 2015). According to Kantak et al. (2017), bimanual movements can be categorized based on symmetry (symmetric or asymmetric) and task goal (independent, parallel or cooperative). In general, it has been well established in decades of research that symmetric tasks are more easily performed than asymmetric ones and thus require less cortical activity [for review see Swinnen (2002)]. The difference between cooperative and non-cooperative tasks is more nuanced. A greater degree of bilaterally organization in terms has been proposed for cooperative movements due to enhanced reflexes and sensory processing bilaterally (Schrafl-Altermatt and Dietz, 2014; Dietz et al., 2015; Schrafl-Altermatt and Easthope, 2018). So far, it is not known but only assumed that descending motor output also exhibits more bilateral organization (Dietz et al., 2015). To answer this question as well as understand the influence task-difficulty on possible modulation of motor output, investigation of ipsilateral primary motor cortex (iM1) excitability in symmetric–asymmetric and cooperative–non-cooperative contexts would provide direct insights.

Activity of ipsilateral M1 during manual tasks is well documented (Ziemann and Hallett, 2001; Cisek et al., 2003; Yarosh et al., 2004; Ghacibeh et al., 2007; Avanzino et al., 2008; Bradnam et al., 2010; Suzuki et al., 2013; Tazoe and Perez, 2014; Giboin et al., 2017). It has been shown that this activity is modulated by different factors such as activation of different muscle groups (Tazoe and Perez, 2014), motor skill acquisition (Zimerman et al., 2014), or recovery after stroke (Bradnam et al., 2013; Bani-Ahmed and Cirstea, 2020). Despite considerable advances in this scientific field in recent years, the functional role of ipsilateral M1 activity in healthy humans is not yet fully understood [for review see Bundy and Leuthardt (2019)]. One reason for this lack of understanding might lie in the fact that most studies use an indirect measure of iM1 activity. A direct measure of iM1 activity can be obtained by analyzing ipsilateral motor evoked potentials (iMEP), but these can be difficult to elicit using standard neurophysiological protocols with transcranial magnetic stimulation (TMS) (Wassermann et al., 1994; Ziemann et al., 1999; Chen et al., 2003; McCambridge et al., 2016). Pre-activation of the target muscles is prerequisite for evoking iMEPs. A significant development for investigating iMEPs was proposed by Schwerin et al. (2011) who used a paired-pulse maximal intensity TMS protocol in stroke-survivors and healthy participants. However, iMEPs were only reliably recorded in stroke patients, where also different protocol parameters had been applied compared to the protocol used in healthy participants. The aim of the present study was twofold: (1) establishing a TMS protocol based on the one used on stroke survivors by Schwerin et al. (2011) to elicit iMEPs in healthy participants, and (2) to investigate the influence of different bimanual tasks on iMEPs and cMEPs elicited by paired-pulse maximal TMS. We hypothesized higher prevalence and increased amplitudes of iMEPs during cooperative compared to non-cooperative bimanual tasks that could not be accounted for by an increase in contralateral M1 (cMEP) excitability. In respect to establishing the TMS protocol, we intended to use paired-pulse stimulations at maximal intensity as described in the previous work (Schwerin et al., 2011) but optimize coil position as well as the interval between the two pulses for iMEP elicitation.

## Materials and methods

### Participants

The study was approved by the University of Auckland Human Participants Research Ethics Committee and was conducted according to the Declaration of Helsinki. Sixteen neurologically healthy, right-handed adults (six females, median age 25 years, range 20–53 years) participated in this study. Each participant gave written informed consent and completed a TMS safety questionnaire.

### Electromyography

Surface electromyography (EMG) was recorded bilaterally from extensor carpi ulnaris (ECU), flexor carpi radialis (FCR), biceps brachii (BBR), and first dorsal interosseous (FDI) using 15 mm^2^ sensor area Ag/AgCl recording electrodes (Ambu, Ballerup, Denmark) positioned over the corresponding muscle bellies. EMG signals were amplified (1000x) using an AMT-8 amplifier (Bortec Biomedical, Calgary, Canada), band-pass filtered (10–1000 Hz) and sampled at 2000 Hz with a CED1401 data acquisition board (CED, Cambridge, UK) and Signal software (Version 6.05; CED, Cambridge, UK).

### Transcranial magnetic stimulation

A MagPro 100 with MagOption stimulator (MagVenture, Denmark) connected to an active-cooled figure-of-eight coil (70 m) was used to deliver monophasic TMS. Participants wore a custom cap with a marked grid starting from the vertex and extending 10 cm to the left, 5 cm to the front and 5 cm to the back in 1 cm increments. They also wore earplugs to prevent hearing damage and startle reflex from the noise of stimulation. First, the optimal site on the left hemisphere to elicit consistent contralateral MEP (cMEP) in the resting right ECU with single-pulse TMS was assessed, as this can be done with single pulse TMS at low intensities. Additionally, some previous studies have shown, that the contralateral hotspot might be ideal for eliciting iMEPs as well (MacKinnon et al., 2004). At this site, paired-pulse TMS at 100% maximal stimulator output (MSO) was delivered in accordance with the protocol used by Schwerin et al. (2011) with different inter-stimulus intervals (ISI) to assess optimal ISI for eliciting ipsilateral MEPs (iMEP) in the left ECU during bilateral isometric wrist extension. The first ISI to be tested was always 30 ms, which was reported to be the optimal ISI in stroke patients (Schwerin et al., 2011) followed by 25 ms. Subsequently, ISI was increased or decreased in steps of 5 ms until optimal ISI was determined. Using paired-pulse TMS with the optimal ISI, the hotspot for iMEP was assessed by moving the coil in steps of 1 cm in all directions starting from the cMEP hotspot. The coil position was marked on the cap. Twenty stimuli were delivered randomly every 8 to 10 s at this spot in each of the task conditions. TMS was triggered by rmsEMG level in left ECU set to 75% of the typical EMG activity during the task. This procedure delivered TMS consistently at the beginning of left wrist extension. Setting the same threshold for all conditions also ensured similar pre-trigger activation between the conditions.

### Setup and movement tasks

**FIGURE 1:**
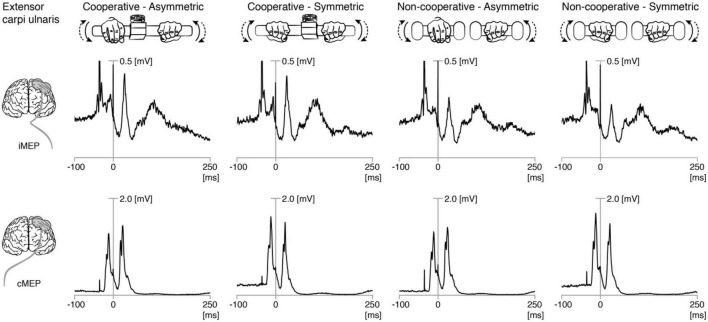
Rectified EMG traces showing motor evoked potentials (MEP) in **left** and **right** extensor carpi ulnaris. Grand averages of rectified EMG showing ipsilateral (iMEP, **top panel**) and contralateral (cMEP, **bottom panel**) MEPs elicited during the four bimanual tasks. The first stimulation (first stimulation artifact) was usually only followed by cMEP. The second stimulation (0 ms) was followed by cMEP and iMEP.

Participants were comfortably seated in a chair with back support and with forearms resting on a pillow placed on the lap. Tasks were performed with outstretched arms in a 90° shoulder flexion position and then to make wrist flexion extension movements bilaterally and rhythmically in time with an auditory metronome at a frequency of 0.75 Hz. Participants were instructed to start with left wrist flexion “on the beat” and continue in the required pattern until TMS was delivered. Stimulation would disrupt the movement and participants were instructed to pause for 3 or 4 beats before re-starting in time with the metronome. Movement resistances were matched to 60% of maximal EMG activity of the left ECU which was measured separately. The non-cooperative movements were performed with dumbbells. A device called ARCO was used for the cooperative tasks. ARCO consists of two handles connected over a shoe-type brake. The handles can be rotated against each other for counteractive movements. The resistance for this movement can be adapted by regulating the force of the brake. Additional weights can be added to the center of ARCO for supporting movements. The four movement conditions are depicted in Figure 1. The cooperative asymmetric task (CA) was a counteractive movement, i.e., the handles of ARCO were rotated against each other by counteractive wrist movements. The cooperative symmetric task (CS) was a supporting movement of the two wrists, i.e., ARCO was moved up and down by extending and flexing both wrists. For the non-cooperative asymmetric task (NA), the left wrist was extended while the right wrist was flexed and vice versa. The non-cooperative symmetric task (NS) consisted of simultaneous extension and flexion of the wrists. During pre-measurements, bilateral isometric wrist extension was performed in the same position holding the same dumbbells later used for the non-cooperative tasks.

### Data analysis

Electromyography data were analyzed using Signal software (Version 6.05; CED, Cambridge, UK). A blinded examiner rated each trial to determine whether an iMEP was present in the left ECU in order to determine iMEP persistence. Participants who had at least four out of 20 iMEPs in each condition were further classified as iMEP^+^ for subsequent analyses. Peak-to-peak amplitudes of iMEP and cMEP were determined for each trial and then averaged for each condition. Signals were then rectified and averages of the 20 trials per condition were calculated for each participant. Pre-trigger RMS EMG (rmsEMG) was calculated between 32 and 2 ms before the first stimulation. Due to the dynamic nature of the task, this relatively short window for pre-trigger RMS had to be chosen. Latencies of all MEPs [iMEP elicited by the second TMS pulse (iMEP_2_), cMEP elicited by the first TMS pulse (cMEP_1_), and cMEP elicited by the second TMS pulse (cMEP_2_)] were manually set. To obtain a measure of excitability (additionally to amplitude) for ipsilateral muscles, the area under the curve (AUC) was calculated over 20 ms starting from individual latency. A measure of inhibition along the crossed pathway was also obtained from the post-MEP silent period (SP). End of SP was defined as the time point where the EMG trace after cMEP_2_ exceeded (rmsEMG)-(2 × SDrmsEMG), with SDrmsEMG calculated as standard deviation of EMG signal in the rmsEMG time window. Amplitude ratios (iMEP_2_/cMEP_2_) were calculated to analyze whether possible iMEP modulation were due to general M1 excitability changes or specific for ipsilateral control. As a measure of refractoriness, the amplitude difference between cMEPs were determined as (cMEP_2_ amplitude)—(cMEP_1_ amplitude). Finally, latency differences between iMEP_2_ and cMEP_2_ and between cMEP_1_ and cMEP_2_ were calculated.

### Statistical analysis

Statistical analyses were performed using SPSS (Version 23; IBM, New York, NY, USA). Normality was assessed using the Shapiro-Wilk’s test and sphericity was assessed using Mauchly’s test. cMEP data was analyzed with two-way repeated measures ANOVA (with Greenhouse-Geisser correction) to determine main effects of SYMMETRY (Symmetric, Asymmetric), TASK (Non-cooperative, Cooperative) and their interaction. Significant effects were addressed *post-hoc* with pair-wise two-sided *t*-tests to check for differences between NS and CS, NA and CA, NS and NA, and CS and CA. Deviations from normality (iMEP data) were handled by using non-parametric tests. Differences between conditions were calculated using Friedman’s tests followed by pair-wise comparisons between the same pairs described above (Wilcoxon’s tests). The significance level was set at *p* < 0.05. *Post hoc* analyses were corrected for multiple comparisons using modified Bonferroni’s procedure (Rom, 1990). Mean and standard deviation (SD) are reported in text unless otherwise stated.

## Results

Paired-pulse TMS at 100% MSO was well tolerated by all participants. Hotspots for iMEP and cMEP were the same for nine participants and radially distributed within 1 cm for the remaining participants. Location of the iMEP hotspot did not explain the observed results, i.e., some iMEP + participants had the same hotspot for iMEPs and cMEPs while other iMEP + participants showed different hotsport of cMEPs and iMEPs. Figure 1 shows the grand average ECU EMG traces from all trials and participants, showing ipsilateral and contralateral MEPs. Nine participants were classified as ECU iMEP^+^ participants. Age and gender of participants could be ruled out as confounding factors in the results.

### Ipsilateral MEPs

Transcranial magnetic stimulation was delivered during left wrist extension, i.e., during dynamic contraction of the left ECU making this the primary muscle of interest. Most results are therefore only presented for iECU.

**FIGURE 2:**
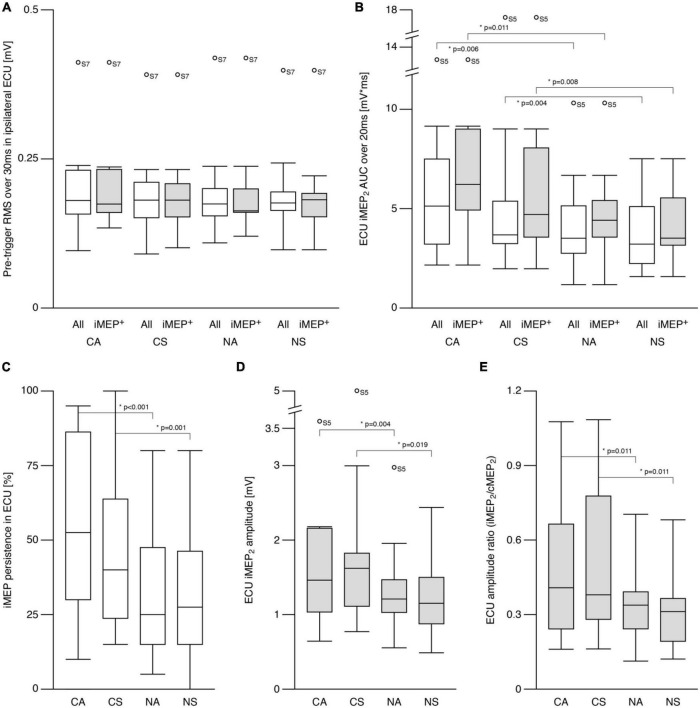
Ipsilateral motor evoked potentials (iMEP) in extensor carpi ulnaris (ECU). **(A)** Root mean squared (RMS) electromyographic signal (EMG) before first stimulation onset was comparable across conditions for all participants as well as for the sub-group of participants showing at least 20% iMEP persistence in every condition (iMEP^+^). **(B)** Area under the curve (AUC) of the iMEP following the second stimulation (iMEP_2_) was greater in cooperative compared to non-cooperative tasks for all participants as well as for iMEP^+^ participants only. **(C)** iMEP persistence was higher in cooperative compared to non-cooperative tasks. **(D)** iMEP peak-to-peak amplitude (only calculated for iMEP^+^ participants) was greater during cooperative compared to non-cooperative tasks. **(E)** Amplitude ratio [iMEP/cMEP (contralateral MEP)] was higher in cooperative compared to non-cooperative tasks. CA, cooperative—Asymmetric task; CS, cooperative—Symmetric task; NA, non-cooperative—Asymmetric task; NS, non-cooperative—Symmetric task. White boxes: all participants, gray boxes: iMEP^+^ participants only, Circles with participant numbers (such as S7): outliers.

The pretrigger rmsEMG of the ipsilateral (left) ECU (iECU) was comparable across all conditions for all participants as well as when including only iMEP^+^ participants (all *p* > 0.5; Figure 2A). ECU iMEP_2_ areas were larger (*Z* = −4.4, *p* < 0.001) during cooperative (5.41 ± 3.4 mV × ms) compared to non-cooperative (3.89 ± 2.0 mV × ms) tasks (Figure 2B).

The persistence of ECU iMEP_2_ was greater (*Z* = −4.6, *p* < 0.001) for cooperative (50.5 ± 28.8%) compared to non-cooperative (31.6 ± 22.1%) tasks. Persistence did not differ between symmetric and asymmetric movements (Figure 2C). For iMEP^+^ participants only, iMEP amplitude (Figure 2D) was larger (*Z* = −2.9, *p* = 0.003) in cooperative (1.60 ± 1.09 mV) compared to non-cooperative (1.12 ± 0.56 mV) tasks. Amplitude ratios (Figure 2E) were higher (*Z* = −3.593, *p* < 0.001) during cooperative (0.49 ± 0.30) compared to non-cooperative (0.34 ± 0.18) tasks.

For FCR rmsEMG, a main effect of TASK was found (*F* = 13.5, *p* = 0.002). *Post hoc* comparisons revealed greater values for CA compared to CS (*t* = −2.6, *p* = 0.019), which is why this effect can be ruled out as a factor when comparing cooperative and non-cooperative tasks For BBR and FDI, no differences in pretrigger rmsEMG were found. Ipsilateral MEPs were also present in FCR and BBR muscles but not in FDI (Supplementary Figure 1).

### Contralateral MEPs

The pretrigger rmsEMG of the contralateral muscles differed between symmetric and asymmetric movements (rECU: *S* = 0.11 ± 0.03 mV, *A* = 0.04 ± 0.02 mV, *F* = 52.7, *p* < 0.001; rFCR: *S* = 0.01 ± 0.01 mV, *A* = 0.03 ± 0.02 mV, *F* = 14.9, *p* = 0.002) but not TASK (All *p* > 0.5). Main effects of SYMMETRY were also found for ECU cMEP_1_ amplitude (*F* = 6.6, *p* = 0.022), ECU cMEP_2_—cMEP_1_ amplitude difference (*F* = 5.5, *p* = 0.033), FCR cMEP_1_ amplitude (*F* = 13.9, *p* = 0.002), FCR cMEP_2_ amplitude (*F* = 7.3, *p* = 0.016), and FCR cMEP_2_—cMEP_1_ amplitude difference (*F* = 9.7, *p* = 0.007) but not ECU cMEP_2_ amplitude (*p* > 0.4; see Table 1).

**TABLE 1:** Contralateral motor evoked potentials.

	ECU			FCR		
	**cMEP_1_ (mV)**	**cMEP_2_ (mV)**	**Amplitude difference (mV)**	**cMEP_1_ (mV)**	**cMEP_2_ (mV)**	**Amplitude difference (mV)**
CA	1.81 ± 0.87	2.07 ± 0.86	0.26 ± 0.46	1.71 ± 0.86	1.66 ± 0.90	−0.05 ± 0.39
CS	2.21 ± 0.73	1.98 ± 0.72	−0.22 ± 0.47	1.06 ± 0.60	1.48 ± 0.71	0.42 ± 0.52
NA	1.86 ± 0.68	2.07 ± 0.80	0.18 ± 0.49	1.79 ± 1.00	1.74 ± 0.89	−0.05 ± 0.48
NS	2.23 ± 0.76	2.06 ± 0.75	−0.17 ± 0.38	1.18 ± 0.62	1.49 ± 0.72	0.31 ± 0.54

cMEP_1_, contralateral motor evoked potential (cMEP) following the first stimulation; cMEP_2_, cMEP following the second stimulation; ECU, extensor carpi ulnaris; FCR, flexor carpi radialis; CA, cooperative—Asymmetric task; CS, cooperative—Symmetric task; NA, non-cooperative—Asymmetric task; NS, non-cooperative—symmetric task.

**FIGURE 3:**
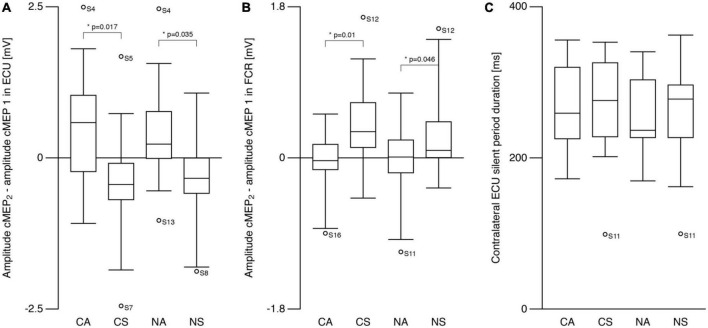
Contralateral motor evoked potentials (cMEP). **(A)** Right ECU cMEP peak-to-peak amplitude difference (see text) differed between symmetric and asymmetric tasks. cMEP_2_ was larger compared to cMEP_1_ during asymmetric tasks and smaller during symmetric tasks. **(B)** Amplitude difference (cMEP_2_–cMEP_1_) in flexor carpi radialis (FCR) was also different between symmetric and asymmetric tasks showing the opposite behavior compared to ECU. cMEP_2_ was smaller compared to cMEP_1_ during asymmetric tasks and larger during symmetric tasks. **(C)** Silent period following cMEP_2_ was similar during all tasks. CA, cooperative—Asymmetric task; CS, cooperative—Symmetric task; NA, non-cooperative—Asymmetric task; NS, non-cooperative—Symmetric task, Circles with participant numbers (such as S4): outliers.

Figures 3A, B show the amplitude differences (cMEP_2_—cMEP_1_) for ECU and FCR, respectively. *Post hoc* analyses revealed differences for SYMMETRY during both TASKS for ECU (CA vs. CS: *t* = −2.7, *p* = 0.017; NA vs. NS: *t* = −2.3, *p* = 0.035) and FCR (CA vs. CS: *t* = 4.1 *p* = 0.01; NA vs. NS: *t* = 2.1, *p* = 0.046). ECU amplitude decreased from cMEP_1_ to cMEP_2_ during symmetric movements and increased during asymmetric movements while the opposite was observed in FCR.

For contralateral silent period (Figure 3C), there were no effects of TASK or SYMMETRY or any interaction (all *p* > 0.6; CA: 270.6 ± 54.5 ms, CS: 269.8 ± 67.6 ms, NA: 259.0 ± 50.2 ms, NS: 262.2 ± 64.8 ms).

### Latencies

**FIGURE 4:**
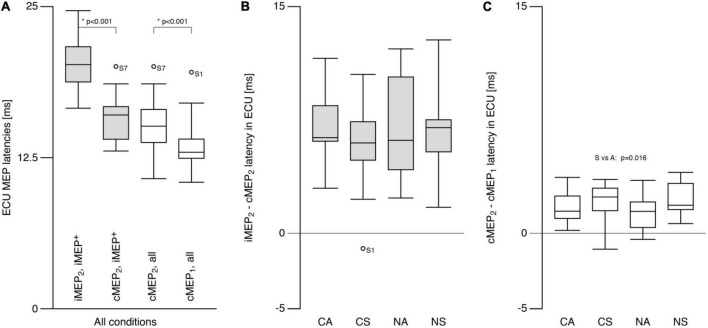
Extensor carpi ulnaris (ECU) motor evoked potential latency. **(A)** Ipsilateral MEP (iMEP, left ECU) had longer latencies than contralateral MEP (cMEP, right ECU) for iMEP + participants (see text). cMEP_2_ had longer latencies than cMEP_1_. **(B)** The difference between iMEP and cMEP latencies was only analyzed for iMEP^+^ participants and did not differ between tasks. **(C)** The difference between cMEP_2_ and cMEP_1_ was smaller for asymmetric compared to symmetric tasks. CA, cooperative—Asymmetric task; CS, cooperative—Symmetric task; NA, non-cooperative—Asymmetric task; NS, non-cooperative—Symmetric task, Circles with participant numbers (such as S7): outliers.

Contralateral motor evoked potential latencies (13.3 ± 1.6 ms) were shorter than cMEP_2_ latencies (15.2 ± 1.8 ms; *t* = −6.8, *p* < 0.001; Figure 4A). iMEP_2_ latencies were longer (*t* = 9.5, *p* < 0.001) than both cMEP latencies, and did not differ between conditions (*p* > 0.7; SN: 20.3 ± 2.0 ms, AN: 20.5 ± 3.2 ms, SC: 20.2 ± 2.6; AC: 19.9 ± 2.4 ms). For iMEP^+^ participants, mean differences between iMEP_2_ and cMEP_2_ latencies were similar across conditions (Mean difference = 6.7 ± 3.1 ms; Figure 4B). A main effect of SYMMETRY (*F* = 7.4, *p* = 0.016) was found for the cMEP latency difference (Figure 4C) with a mean difference of 2.2 ± 1.1 ms for symmetric conditions and 1.5 ± 1.0 ms for asymmetric conditions.

## Discussion

The present study investigated changes in corticomotor excitability through direct crossed and indirect uncrossed descending pathways during different types of bimanual movements. The main findings were that: (1) iMEPs could be elicited with the paired-pulse technique in forearm muscles in healthy participants, (2) iMEP persistence and size increased during cooperative compared to non-cooperative bimanual movements, and (3) cMEPs were modulated by the symmetry of the movement.

### Common goal vs. dual-goal movements

A key dimension along which bimanual movements are characterized is whether or not the two hands work toward a common goal or act independently of one another (Kantak et al., 2017). Common goal tasks can further be divided into cooperative and parallel movements. In the present study, the cooperative task reflected a common goal, whereas the non-cooperative task reflected a dual-goal task where each hand was moved independently. Nevertheless, dual-goal movements are usually spatially and temporally coupled leading to similar amplitudes, rhythms and even forces (Bogaerts and Swinnen, 2001; Swinnen et al., 2001; Diedrichsen et al., 2003). In the present study, the protocol to was designed to minimize confounds from these latter aspects across the four tasks. There are known differences in neuromotor control between common and dual-goal tasks. For example, intracortical inhibition is reduced in dual-goal tasks compared to common-goal tasks (Liao et al., 2018). Cooperative movements are more likely to be under bilateral neural control than non-cooperative movements made with the two hands. In the present study, we observed an increase in both the persistence of elicited iMEPs and the size of iMEPs for cooperative compared to non-cooperative tasks. This fits well with previous findings that cooperative hand movements rely on more bilaterally organized neural control. For example, electrical stimulation of the ulnar nerve produces EMG reflex responses on both arms only during cooperative, but not during non-cooperative movement (Dietz et al., 2015; Schrafl-Altermatt and Easthope, 2018). Where along the neuroaxis the contralateral reflex responses are generated has been unclear. The current results lead us to suspect that cooperative hand movements rely on enhanced descending excitatory projections from both hemispheres. This task-specific up-regulation during complex cooperative movements of the upper limbs reveal a functional relevance of ipsilateral motor control in healthy participants.

### Symmetric vs. asymmetric bimanual movement

The symmetry of bimanual coordinated movement, i.e., the hands moving in-phase or anti-phase to each other, did not influence the persistence or size of the iMEP. A previous report found increased iMEP size in biceps brachii during isometric activation of contralateral triceps compared to activation of contralateral biceps (Tazoe and Perez, 2014). The authors interpreted this finding as evidence for selective disinhibition which engaged ipsilateral motor pathways. An up-regulation of ipsilateral pathway excitability during homologous movements is in line with the idea that the default mode for upper limb movement is mirror symmetric bimanual movement that is then selectively inhibited (Kagerer et al., 2003; Grefkes et al., 2008; Byblow et al., 2012; Watanabe et al., 2017). Since inhibition is unnecessary for mirror-symmetric bimanual movement, up-regulation of the ipsilateral hemisphere could be expected. By contrast, an effect of task symmetry was observed contralaterally. In ECU, the cMEP amplitude (from the second stimulation) was larger than cMEP_1_ amplitude during asymmetric tasks but the opposite pattern of modulation was observed for symmetric tasks. Using a similar paired-pulse paradigm (Schwerin et al., 2011) there was an increase in the 2nd cMEP amplitude compared to the 1st. Part of our results can be explained by the differences in the pre-activation. Since TMS was always delivered during extension of the left ipsilateral wrist, the right contralateral extensors were only active during symmetric movements. Naturally cMEP_1_ in ECU was therefore larger during symmetric compared to asymmetric tasks. However, amplitudes of cMEP_2_, which are elicited during the silent period after the first cMEP, remained similar between tasks. Whether this represents a ceiling effect of the second cMEP or reflects a task symmetry effect remains unclear.

### iMEP characterizations

In the present study, iMEPs have been elicited in wrist extensor muscles in the majority of the included healthy participants. We could classify 9 out of 16 participants as iMEP+. This number is lower compared to other studies; Tazoe and Perez (2014) for example reported almost 75% of participants being iMEP+. However, thy measured iMEPs in more proximal muscles, which are known to be more bilaterally innervated. So far, there is no study showing reliable iMEP activity in forearm muscles in healthy participants. Schwerin et al. (2011) were able to show similar iMEP persistence in distal muscles of stroke patients. Taking these factors into consideration, we argue that both the iMEP + rate as well as the persistence of iMEPs shown in the present study are to be considered as remarkably high.

In this study, only the second stimulation of the paired-pulse protocol was usually followed by an iMEP. Facilitation of MEPs with paired-pulse protocols using ISIs between 10 and 50 ms have been shown before on contralateral side (Abbruzzese et al., 1999; Schwerin et al., 2011). Enhanced I-waves using suprathreshold conditioning stimulus 25 ms before test stimulus lead to the conclusion that the facilitation is at least partially of cortical origin (Nakamura et al., 1997) and could thus be generalized also for ipsilaterally projecting neurons. A possible mechanism is NMDA and AMPA receptor mediated excitatory post-synaptic currents (EPSC), which are enhanced by paired-pulse stimulations with ISIs between 25 and 100 ms (Clark et al., 1994). In their study, NMDA EPSC was enhanced only when the cells were hyperpolarized. Further studies will be required to determine whether an iMEP facilitation is associated with an iSP after single pulse stimulation. Temporal overlay of second stimulation with disinhibition from the rebound period after the iSP might also add to the facilitatory effect of the paired-pulse protocol.

Higher activation of the right compared to left hemisphere (in right handers) has been shown in the motor areas during common goal tasks, as well as impaired coordination when virtual lesions are applied to these same areas (Duque et al., 2010). Conversely, the left hemisphere has been shown to be more involved in ipsilateral movement control compared to the right one (Ziemann and Hallett, 2001; Ghacibeh et al., 2007; Suzuki et al., 2013). For these reasons, we decided to target the left hemisphere to increase the possibility of eliciting iMEP in the present study. Also for right-handers, the dominant left hemisphere is known to control bimanual movements more than the non-dominant right hemisphere (Serrien et al., 2003; Stinear and Byblow, 2004). Despite these findings we cannot know from the present study the possible effects that may have been observed on iMEPs had the right hemisphere been stimulated. This topic awaits further investigation.

Optimal coil placement for iMEP and cMEP was the same for the majority of participants and only deviated slightly for others in keeping with a previous finding (MacKinnon et al., 2004) and in contrast to studies that have found iMEP hotspots anterio-lateral of the cMEP hotspots (Wassermann et al., 1994; Kagerer et al., 2003). The finding may be attributable to using maximal stimulation intensities and a paired-pulse protocol which will reduce the focality of stimulation compared to lower intensity, single-pulse application.

The latency difference between cMEP and iMEP was on average 6.5 ms which is in keeping with previous findings (Ziemann et al., 1999; MacKinnon et al., 2004). The long latency relative to cMEPs, and the fact that iMEPs can be modulated by head rotation (Ziemann et al., 1999), indicate that the iMEP may reflect, at least in part, descending activity along the cortico-reticulo-spinal pathway (Wassermann et al., 1994; Ziemann et al., 1999).

It has been debated whether the reticulo-spinal tract (RST) projects onto alpha moto-neurons innervating distal arm and hand muscles in primates and humans (Lemon, 2008). Direct evidence from monkey studies has indicated that hand and wrist alpha motoneurons receive mono- and di-synaptic input from the RST (Riddle et al., 2009). The projections from RST onto distal alpha motor neurons were as strong and common as those onto more proximal areas. Additionally, bimanual movements in monkeys could only be explained by activity in either the cortico-spinal or the reticulo-spinal projections alone to about 40%, whereas 45% was explained by an interaction of the two (Ortiz-Rosario et al., 2014). In humans, there is only indirect evidence for RST contributions. For example, using an acoustic startle paradigm, it has been shown that the “StartReact response” is mediated by the RST, and is present during both finger and hand movements made in a reaction time context (Honeycutt et al., 2013; Baker and Perez, 2017). Overall there is good reason to think that the RST innervates human wrist extensor muscles and is a key pathway transmitting the iMEP.

An up-regulation of RST activity has been shown during coordinated hand tasks compared to individuated finger tasks (Honeycutt et al., 2013). Further up-regulation by increasing the degree of coordination necessity from one hand to two hands, and from non-cooperative to cooperative movements may be responsible for further up-regulation along the RST.

### Potential clinical relevance

It has often been proposed that ipsilateral or bilateral pathways may play a role in upper limb motor control when the crossed corticospinal pathway is injured, such as after stroke (Jankowska and Edgley, 2006; Baker, 2011; Bradnam et al., 2013). While there is evidence for up-regulation of ipsilateral motor cortical activity during movements of the paretic arm, it is still not clear whether this increased activity is beneficial [for review see Buetefisch (2015)]. Similar studies have observed a negative relationship between ipsilateral activity and functional impairment (Serrien et al., 2004; Teasell et al., 2005). However, a recent study showed larger improvements during training in patients with higher prevalence of iMEP (Hammerbeck et al., 2019). In previous studies, ipsilateral pathways have usually been studied in a non-functional context in post-stroke participants. Here we show for the first time that cooperative movements enhance ipsilateral output and could create the functional context for these pathways. This idea complements recent findings which found more pronounced influence of the contralesional hemisphere over the paretic arm in stroke patients during cooperative movements (Schrafl-Altermatt and Dietz, 2016a,b). It remains to be seen whether this movement context can be exploited to improve recovery of upper limb function, but the results of the present study certainly support the idea of introducing bimanual, cooperative training in rehabilitation programs for stroke patients.

### Limitations

The present study has limitations. First, modulation of ipsilateral responses to single pulse TMS has not been analyzed. Ipsilateral silent period as well as rebound activity might be influenced by task-goal and contribute to the differences found in iMEP persistence and size. Second, only the left (dominant) hemisphere was stimulated. Additionally, iMEP persistence was on average between 25 and 50% despite the paired-pulse protocol, which we consider a high persistence for this measure in forearm muscle of healthy participants who have not been pre-selected but might still limit the conclusions that can be drawn from the results.

## Conclusion

Ipsilateral projections to proximal as wells distal muscles can be tested in healthy participants with the further optimized paired-pulse TMS protocol that has been established in the present paper. It thus serves a novel and interesting tool for examination of ipsilateral motor pathways. The excitability of these connections is up-regulated during common-goal cooperative tasks showing a functional relevance of ipsilateral motor control in bimanual coordination. These results add to the understanding of upper limb motor control and how more complex tasks such as bimanual cooperative movements require modulation of descending output in order to accomplish coordination of force and timing between the two sides. Additionally, the results provide a reasoning for application of bimanual, cooperative training in post-stroke rehabilitation.

## Data availability statement

The raw data supporting the conclusions of this article will be made available by the authors, without undue reservation.

## Ethics statement

The studies involving human participants were reviewed and approved by the University of Auckland Human Participants Research Ethics Committee. The patients/participants provided their written informed consent to participate in this study.

## Author contributions

MA: design and conduct of the study, data analysis, and writing of manuscript. HJ and KH: conduct of the study and revising of manuscript. WB: design of the study and revising of the manuscript. All authors contributed to the article and approved the submitted version.
