# Supplementary material for: Modulation of ipsilateral motor evoked potentials during bimanual coordination tasks
Source: Front Hum Neurosci. 2023 Sep 6;17:1219112. doi: 10.3389/fnhum.2023.1219112 (PMC10509758; doi:10.3389/fnhum.2023.1219112)
Supplement: Supplementary file 1 [file Image_1.pdf]

Supplement

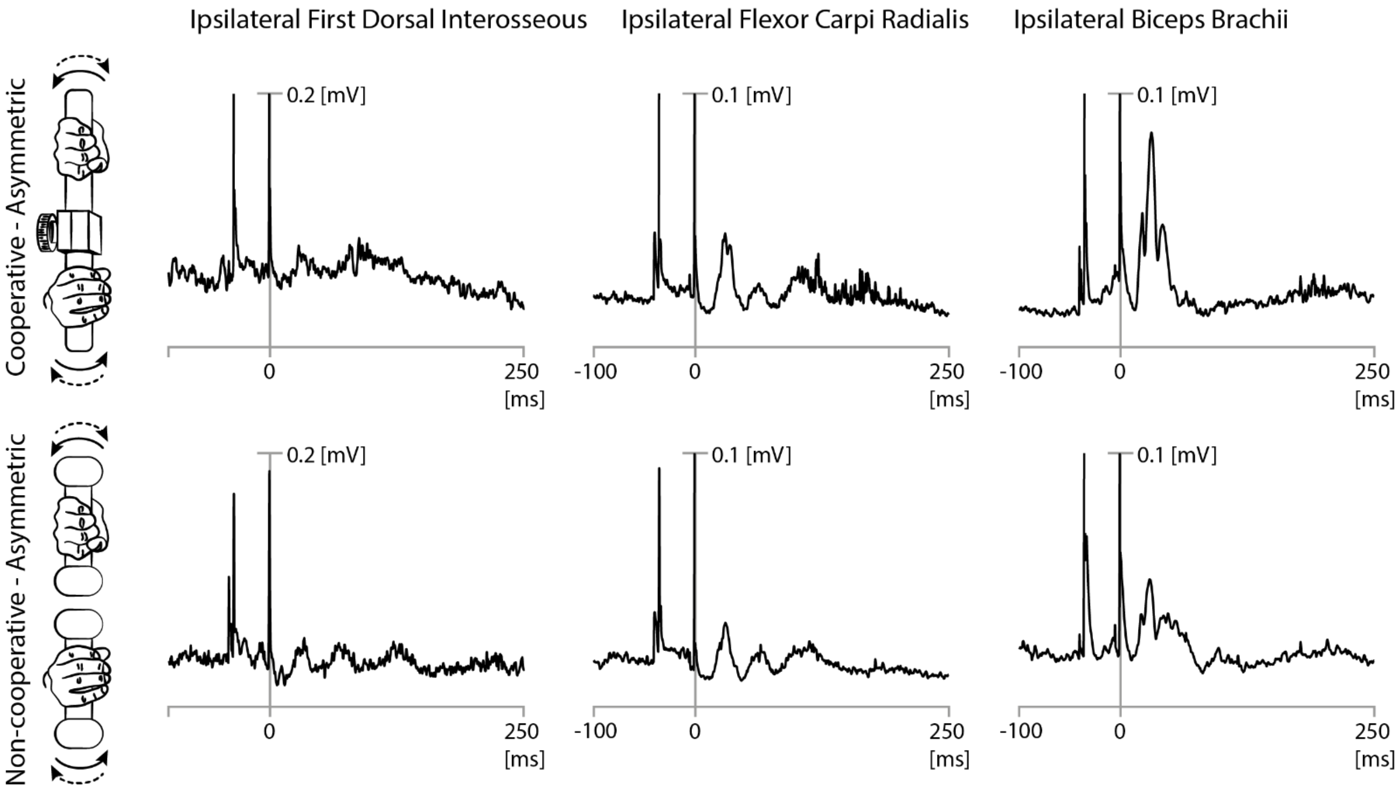

Figure S1: Rectified EMG traces showing ipsilateral motor evoked potentials (iMEPs).

Grand averages of rectified EMG measured over first dorsal interosseous (FDI), flexor carpi radialis (FCR) and biceps brachii (BBR) showing iMEPs elicited during a cooperative (upper panels) and a non-cooperative (bottom panels) bimanual task.
